# Briquettes Made of Branches Wood of Three Mangrove Species Bonded by Starch Adhesive

**DOI:** 10.3390/ma16155266

**Published:** 2023-07-27

**Authors:** Hardiansyah Tambunan, Arif Nuryawan, Apri Heri Iswanto, Iwan Risnasari, Mohammad Basyuni, Widya Fatriasari

**Affiliations:** 1 Faculty of Forestry, 2nd Campus in Kuala Bekala, Universitas Sumatera Utara, Medan 20355, Indonesia; htambunan911@gmail.com (H.T.); apri@usu.ac.id (A.H.I.); iwan1@usu.ac.id (I.R.); m.basyuni@usu.ac.id (M.B.); 2 Center Excellence for Mangrove (PUI Mangrove), Universitas Sumatera Utara, Medan 20155, Indonesia; 3 Research Center for Biomass and Bioproducts, National Research and Innovation Agency (BRIN), Jl Raya Bogor KM 46 Cibinong, Bogor 16911, Indonesia; widy003@brin.go.id

**Keywords:** wood briquettes, biomass energy, renewable energy, mangrove species, starch adhesive

## Abstract

The development and utilization of wood briquettes is one of the efforts to reduce dependence on fossil fuels, including to fulfill overseas market need. This study aimed to evaluate the properties of wood briquettes made of the branches of three mangrove species and to analyze the effect of different wood species of mangrove branches, and the types of starch adhesive, on the quality of wood briquettes. The wood briquettes made in this study were 3 cm × 4 cm in a cylindrical shape using three wood species of mangrove branches, namely mata buaya (*Bruguiera sexangula*), buta-buta (*Excoecaria agallocha*), and bakau minyak (*Rhizophora apiculata*), while the adhesives used were tapioca starch, maize starch, and potato starch. The results showed that the moisture, ash content, and calorific value of the wood briquettes mostly met the ISO 17225-3:2-2020 class A2 standard and the specification and quality standards of wood briquettes for Grade A2 issued by the Korea Forest Research Institute, except the density. Wood briquettes made of mata buaya by using the three types of starch adhesives generally had better properties than all other types of wood briquettes. The interaction of mangrove wood species and the types of starch adhesive had a significant effect on the properties of wood briquettes, except for volatile matter and calorific value for which they had no significant effect. The use of wood briquettes from mangrove wood branches contributes to sustainable forest management and maintains the ecological function of mangrove forests while providing environmentally friendly alternative energy for households as a source of fuel/energy. Furthermore, future research is needed, such as investigating the optimal pressing pressure needed to achieve higher density of the wood briquettes.

## 1. Introduction

The use of common energy derived from fossil fuels is increasing in line with the growing population. For instance, in Indonesia, the current pattern of global energy consumption is still dominated by fossil fuels such as coal, gas, and oil. The total final energy consumption in 2021 reached 123 million tons of oil equivalent (MTOE), consisting of 44.2% in the transportation sector, 33.5% in the industrial sector, 16.3% in households, and 6% in the commercial sector and other sectors [1]. However, since fossil fuels are an unrenewable source of energy that takes a very long time (hundreds of thousands of years) to form and can be rapidly depleted, as well as having finite known reserves [2], it is necessary to consider the long-term consequences and sustainability of relying heavily on limited energy sources by diversifying energy sources. This involves developing and implementing alternative energy sources to reduce dependence on fossil fuels. One of the alternative energies that can be utilized is renewable energy sources from biomass [3]. 

Agricultural, plantation, and other plentiful lignocellulosic materials are potential sources of biomass, but they are not being employed in large quantities as an energy source [4]. Forestry biomass is a source of biomass that is abundant and easy to obtain, yet it has not been used optimally as an energy source. This type of biomass can come in various forms, including wood biomass, sawdust, and wood chips from harvesting activities, sawmills, and woodworking industries [5]. 

Currently, woody biomass represents the primary and plentiful natural resource utilized for the creation of various energy types. The benefits of using woody biomass as a raw material for energy are reduced use of fossil fuels and reduced carbon emissions [6]. Wood is utilized as a raw material to generate energy both in traditional forms such as firewood and in forms that undergo further processing to facilitate use, packaging, storage, and transportation, such as pellets and briquettes [7]. Briquettes are defined as solid biofuel made with or without additives in the form of cubiform or cylindrical units with a diameter of over 25 mm produced by compressing pulverized woody biomass [8]. 

Biomass and biowastes have remained a significant alternative to produce sustainable heat and biofuels, not only in emerging countries but also in developed countries. This is mostly due of its naturally advantageous characteristics, such as low production costs, low emissions, and independence from seasonal or weather variations, making energy production sustainable [9]. Additionally, the utilization of biomass for bioenergy benefits nations economically by boosting GDP, particularly when cutting-edge biomass handling and processing technology are used [10]. However, in South Korea these products are still imported, and the country’s wood market is worth about 3.5 billion KRW annually [11]. Other challenges, including supplying rural areas with the sustainable energy they need, ensuring energy security, reducing emissions, halting the loss of forests, reducing air pollution, having a minimal negative impact on the environment and human health, and socioeconomic-related issues have maintained interest in renewable energy research and use [12].

Indonesia has the potential of mangrove forest resources with various benefits, particularly as a source of woody biomass. Even though the most crucial role of mangrove forests is in providing the ecological support [13], the forests also have economic functions, such as fishery production, ecotourism [14], providing a source of wood biomass as construction poles and providing firewood as solid fuel [15]. 

Utilizing firewood obtained directly from the main stem of mangrove trees causes mangrove forests to degrade if the harvesting rate is faster than the growth rate [16,17]. Therefore, efforts can be made by utilizing mangrove woody biomass derived from non-main stem parts, such as branches. In this study, the branches wood was converted into wood briquettes as they are more uniform in shape and size compared to those of firewood, making them easier to stack, transport, and store [18]. Woody biomass from mangrove forests has a high calorific value [19], and by utilizing mangrove woody biomass in the form of branches, the ecological function of the mangrove forest remains protected.

The production of wood briquettes is a prospective effort in processing mangrove wood biomass in the form of branches as a source of solid energy. Briquetting provides added value to mangrove wood branches, can increase calorific value and density, and is cheap and easy to produce, pack, and distribute [20]. Wood briquettes are a fuel obtained by converting wood into sawdust and mixing it with an adhesive, then compacting it without the carbonization process [21]. Wood briquettes are the cheapest energy source produced with the simple technology and equipment used, so it is possible to develop and produce large quantities in a relatively short time.

The mangrove forest in Lubuk Kertang Village, West Brandan District, Langkat Regency, North Sumatra, Indonesia was the location for collecting mangrove wood branches in this study. It has an area of 410 hectares with an IUPHKm (Community Forest Utilization Business Permit) based on SK.987/Menlhk-PSKL/PKPS/PSL.0/3/2017, managed by the Mangrove Farmer and Fisherman Group. The mangrove forest has great potential for the supply of wood branches to produce wood briquettes as an effort to develop and utilize biomass energy. With around 4000 trees per hectare [22] and each tree taken with 3−4 branches, it can produce around 12,000–16,000 branches per hectare.

In this study, starch was used as an adhesive for wood briquettes because starch is an edible polymer and nontoxic [23], possesses high heating value, is widely available, low cost, has good combustion performance, good binding performance, and high mechanical strength [24,25], making it is safe for cooking purposes. Apart from starch adhesives, there are other commonly used adhesives in wood briquette production, such as inorganic adhesives (clay, limestone, cement, sodium silicate, and calcium oxide) and compound adhesives comprising both organic and inorganic adhesives. When compared to these adhesives, starch adhesives offer several advantages. 

Research on the utilization of mangrove wood branches and variations of starch adhesives in the production of wood briquettes has not been carried out before. Previous research has only focused on the basic properties of mangrove wood branches. Based on that, research on making wood briquettes from mangrove branch wood is necessary to find an alternative to fossil fuel energy and to add value to mangrove branch wood as a source of energy in the form of wood briquettes. This study aimed to evaluate the characteristics of wood briquettes made from mangrove wood branches and to analyze the effects of different wood species of mangrove branches and the types of starch adhesive on the properties of wood briquettes.

## 2. Materials and Methods

### 2.1. Materials

Three species of mangrove wood in the form of branches, namely mata buaya (*Bruguiera sexangula*), buta-buta (*Excoecaria agallocha*), and bakau minyak (*Rhizophora apiculata*), were obtained from Lubuk Kertang Village, Brandan Barat District, Langkat Regency, North Sumatra, Indonesia. The adhesives used were tapioca starch adhesive obtained from Bumi Kencana Flour Company (Tangerang, Indonesia), corn starch purchased from Bake King Chemical (Medan, Indonesia), and potato starch produced by Wadah Pangan Makmur Company (Sidoarjo, Indonesia). After synthesizing into starch adhesives, each type of starch adhesive has a solid content of 4.61%, 7.25%, and 8.09%, respectively.

#### 2.1.1. Preparation of Mangrove Wood Raw Materials

The mangrove trees were selected based on the predetermined species that were approximately 10 years old (based on the year planted), and then the branches were directly harvested using a machete. Even though the assessment of moisture content has been conducted according to ASTM D 4442-92 (2003) [26], only results of the density and specific gravity measurements based on ASTM D 2395-14 (2014) [27] are presented in Table 1. 

The branches of the mangrove wood, which were obtained and sorted by species, were then air-dried. Then, they were ground into powder using a hammer mill and passed through a 40-mesh sieve. The powder that passed through the 40-mesh sieve was used as the raw material for wood briquettes, which were then dried under sunlight and oven-dried until a moisture content of <10% was obtained.

#### 2.1.2. Adhesive Production

The starch adhesive was produced by mixing starch and water in a 1:10 (*w*/*w*) ratio [28,29], then heating and stirring until homogeneous and the color changed from white to clear appearance. Before mixing, the particle size of the three species of mangrove wood branch powder and the starch adhesive in gel form were measured using a Particle Size Analyzer (PSA Anallysete 22 Nanotec, Fritsch, Germany) by dispersing the particles in a liquid medium to prevent agglomeration.

#### 2.1.3. Wood Briquettes Production

The wood briquettes produced in this study were 3 cm × 4 cm (diameter × height) following the briquette-making equipment registered patent process [30] and using starch adhesive with a target density of 0.9 g/cm^3^ for five replications. Mixing branch mangrove sawdust with starch adhesive was performed manually in a bucket until evenly distributed. The adhesive content used was 5% based on oven-dry wood sawdust. Mangrove branch sawdust and adhesive that had been mixed evenly were put into briquette-making equipment and the material was subjected to compression through a hydraulic press machine at a pressure of 30 kgf/cm^2^. The wood briquettes obtained were then dried by being exposed to sunlight for 3 days.

#### 2.1.4. Evaluation of the Properties of Wood Briquettes

Determination of the quality of wood briquettes was conducted through physical properties testing of moisture content and density, mechanical properties (compressive strength), chemical properties consisting of ash content, volatile matter, fixed carbon, and calorific value, as well as thermal properties. The properties of wood briquettes from the test results were standardized, referring to ISO 17225-3:2-2020 [31] with a target of class A standard. For accommodating export quality, the specification and quality standards of wood briquettes for Grade A2 issued by the Korea Forest Research Institute (KFRI, Korea Forest Service, Daejeon, Republic of Korea) were also applied for comparison [12].

### 2.2. Physical Properties Testing

#### 2.2.1. Moisture Content

Part of a wood briquette weighing 1 g was placed into a porcelain cup. Then, it was exposed to a temperature of 105 °C for 1 h inside an oven. After that, it was allowed to cool in a desiccator and then weighed immediately upon reaching room temperature. Determination of moisture content referred to the ASTM D 3173-03 standard [32], calculated using the following Formula (1).

(1) $$\mathrm{Moisture}\;\mathrm{Content}=\frac{\mathrm{initial}\;\mathrm{weight}\;\mathrm{of}\;\mathrm{briquettes}\;-\;\mathrm{final}\;\mathrm{weight}\;\mathrm{of}\;\mathrm{briquettes}}{\mathrm{the}\;\mathrm{initial}\;\mathrm{weight}\;\mathrm{of}\;\mathrm{briquettes}}\;\times \;100\%$$

#### 2.2.2. Density

Wood briquettes were weighed using a digital scale to determine their weight. Furthermore, the dimensions of wood briquettes (diameter × height) were measured to determine their volume. Density determination was carried out at a specific moisture content (based on the results of calculating the moisture content of ASTM D 3173-03 standard [32]) and referred to ASTM D 2395-14 standard [27], calculated using the following Formula (2).

(2) $$\mathrm{Density}=\frac{m\;}{v}$$

where m is mass of wood briquettes (g) and v is volume of wood briquettes (cm^3^).

### 2.3. Mechanical Properties Testing

**Figure 1:**
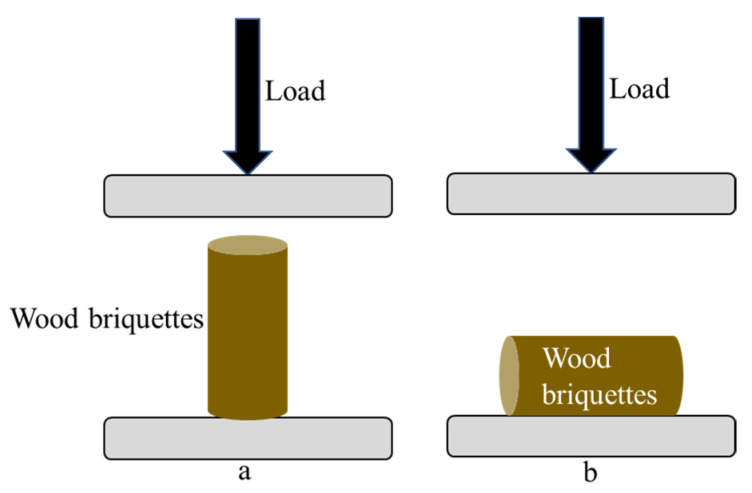
Axial (**a**) and diametral (**b**) compressive test configurations.

Compressive strength refers to the maximum load that a wood briquette can withstand until it breaks or fractures. Compressive strength describes the load wood briquettes can withstand during transportation, handling, and storage [33]. The compressive strength of wood briquettes was evaluated using a universal testing machine (UTM Tensilon RTF-1350, A&D Company, Tokyo, Japan) with a speed of 1 mm/min. Wood briquettes were tested for compressive strength in two directions, namely axial and diametral (Figure 1). Determination of axial compressive strength referred to ASTM D 2166-00 standard [34], which was calculated using the following Formulae (3)–(5).

(3) $$\epsilon =\frac{\Delta L}{L_{0}}$$

(4) $$A=\frac{A_{0}}{\left(1-\epsilon \right)}$$

(5) $$\sigma =\frac{P}{A}$$

where ϵ is axial strain, ΔL changes in length/deformation of wood briquettes (cm), L_0_ is the initial length of wood briquettes (cm), and A_0_ is the initial area of the cross-section of the wood briquettes (cm^2^), A is the cross-sectional area of wood briquettes at a given load (cm^2^), P is given the applied load (kgf), and σ is axial compressive strength (kgf/cm^2^).

Determination of diametral compressive strength referred to ASTM D 3967-95a standard [35], calculated by the Formula (6) as follows.

(6) $$\sigma =\frac{2P}{\pi LD}$$

where σ is diametral compressive strength (kgf/cm^2^), P is given applied load (kgf), L is the length of wood briquettes (cm), and D is the diameter of wood briquettes (cm).

### 2.4. Chemical Properties Testing

#### 2.4.1. Ash Content

The porcelain cup and lid were weighed first, then 1 g of wood briquette with specific moisture content (based on the calculation of moisture content from ASTM D 3173-03 standard [32]) was put into the cup and immediately covered. Then, the lid was removed, and the cup containing the wood briquette sample was placed into a furnace (Thermolyne Furnace Benchtop Muffle Type 48000, Barnstead Thermolyne, Ramsey, MN, USA) and heated gradually from 450 to 750 °C for 4 h. The cup was then taken out of the furnace, covered, cooled, and weighed. The calculation of the ash content was based on the ASTM D 3174-02 standard [36] and was determined using the Formula (7).

(7) $$\mathrm{Ash}\;\mathrm{Content}\;=\frac{\left(A\;-\;B\right)}{C}\;$$

where A is the weight of the porcelain cup + lid + ash (g), B is the weight of the empty porcelain cup + lid (g), and C is the weight of the wood briquette used (g).

#### 2.4.2. Volatile Matter

The porcelain cup and lid were weighed first, then a 1 g part of wood briquette with specific moisture content (based on the calculation of moisture content from ASTM D 3173-03 standard [32]) was put into the cup and closed tightly so that the carbon deposits from the wood briquette sample was not burned. Then it was placed directly into the furnace (Thermolyne Furnace Benchtop Muffle Type 48000, Barnstead Thermolyne, Ramsey, MN, USA) at a temperature of 950 °C for 7 min. The cup was taken out of the furnace and weighed as soon as it cooled. The volatile matter was determined based on the ASTM D 3175-07 standard [37], which was calculated using Formulae (8) and (9) as follows.

(8) $$\mathrm{Weight}\;\mathrm{loss}\;=\frac{\left(A\;-\;B\right)}{A}\;\times \;100\%$$

(9) Volatile matter=C − D

where A is the weight of the wood briquette used (g), B is the weight of heated wood briquette (g), C is weight loss (%), and D is moisture content (%).

#### 2.4.3. Fixed Carbon

The determination of fixed carbon referred to the ASTM D 3172-89 [38] standard, calculated by the following Formula (10).

(10) Fixed Carbon=100% − (moisture content+ash content+volatile matter)

#### 2.4.4. Calorific Value

The Parr Bomb Calorimeter 6400 (Parr Instrument Company, Moline, IL, USA) was utilized to determine the calorific value of wood briquettes by burning wood briquettes in a high-pressure oxygen atmosphere. Determination of calorific value was based on ASTM D5865-10a standard [39]. 

### 2.5. Thermal Properties Testing

Thermogravimetric Analysis (TGA 4000, PerkinElmer, Waltham, MA, USA) was used to evaluate the thermal properties of wood briquettes by preparing wood briquette samples according to their types in the form of powder. TGA is one of the thermal analysis methods for a material, in this case, wood briquettes. It measures the decrease in mass as the material undergoes heat treatment under certain atmospheric conditions. TGA analysis is used to determine the decomposition of the material due to temperature changes [40,41]. The analysis was carried out by heating about 10 mg of wood briquette sample from room temperature (25 °C) to 750 °C at a heating rate of 10 °C/min in a room with nitrogen gas at a flow rate of 20 mL/min during analysis to prevent oxidation. 

### 2.6. Data Analysis

The data obtained from testing the properties of wood briquettes were analyzed statistically with an experimental design using a factorial Completely Randomized Design (CRD) consisting of two factors: factor (A) wood species of mangrove branch consisting of three species of branches wood, namely mata buaya (*Bruguiera sexangula*), buta-buta (*Excoecaria agallocha*), and bakau minyak (*Rhizophora apiculata*); and factor (B) type of starch adhesive comprising three varieties of starch, namely tapioca starch, maize starch, and potato starch with five replications. A Duncan multiple range test (DMRT) was conducted to determine if the factors had a statistically significant effect at a *p*-value < 0.05.

## 3. Results and Discussion

### 3.1. Particle Size Analysis

**Figure 2:**
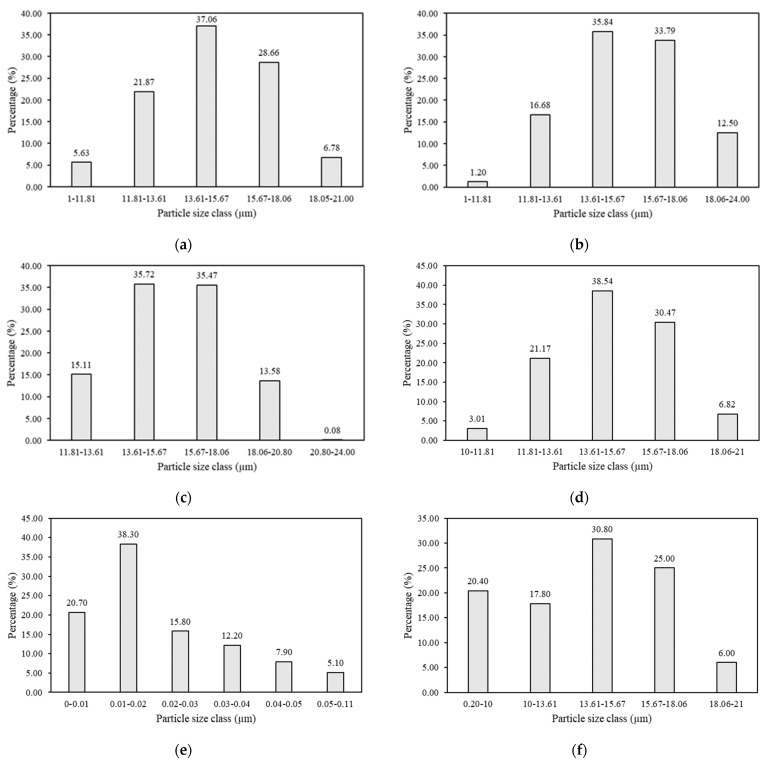
Particle size distribution of mangrove wood sawdust and starch adhesive: (**a**) mata buaya, (**b**) buta-buta, (**c**) bakau minyak, (**d**) tapioca, (**e**) maize, and (**f**) potato.

The particle size distribution of three species of mangrove wood branch sawdust and starch adhesive, obtained by measuring using a PSA with a measurement range of 0.01–42.30 μm, is presented in Figure 2. It is known that the three wood species of mangrove branch sawdust have the same three dominant particle size classes, namely in the 11.81–13.61 µm, 13.61–15.67 µm, and 15.67–18.06 µm size classes. The smallest average particle size distribution was found in the mata buaya sawdust. In contrast, the largest was found in the bakau minyak sawdust. The first had a size of 14.81 µm and the latter was 15.74 µm. Differences in the average particle size distribution of wood species can be caused by the wood properties, especially the density and hardness of the wood. Wood species that are softer and have lower density produce larger/coarse particles, while wood species that are harder and have higher density produce smaller/fine particles [42]. In addition, further processing of wood branches, such as crushing/grinding, can lead to the release of sawdust with different particle size distributions [43]. According to our findings in this study, mata buaya wood species had a high density with a smaller particle size distribution.

Based on Figure 2, the three types of starch adhesives had three different predominant particle size classes. Both tapioca and potato starch adhesives had three similar dominant particle sizes grouped at 11.81–13.61 µm (21.17%), 13.61–15.67 µm (38.54%), and 15.67–18.06 µm (30.47%) for the first and at 0.20–10 µm (20.40%), 13.61–15.67 µm (30.80%), and 15.67–18.06 µm (25.00%) for the latter. Maize starch adhesive also had three predominant particle size classes but relatively smaller compared to ones at 0–0.01 µm (20.70%), 0.01–0.02 µm (38.30%), and 0.02–0.03 µm (15.80%). The smallest average particle size distribution was found in corn starch adhesive, while the largest was found in tapioca starch adhesive, at 0.03 µm and 15.08 µm, respectively. Even though the maize starch had the smallest particle size compared to those of both tapioca and potato, when they were converted into adhesives, the solid content of the adhesives followed the order of tapioca starch (4.61%) < maize starch (7.25%) < potato starch (8.09%), as aforementioned in the materials section. One of the most essential factors connected to interparticle bonding is particle size, which is an indicator of good-quality briquettes. When the particle size of mangrove wood branch sawdust and starch adhesive is smaller, the area and surface contact between the particles increases, thereby increasing the interparticle bonding during the production of wood briquettes [44]. The increase in area and surface contact facilitates the formation of strong bonds between particles and, therefore, improves the strength and density of wood briquettes [45]. Consistent with the density of wood briquettes produced in this study, the smaller particle size distribution of the mata buaya wood species resulted in higher density wood briquettes than buta-buta and bakau minyak wood species. Similarly, the use of maize starch adhesive with a smaller particle size distribution generally produced wood briquettes with higher density compared to tapioca and potato starch adhesives.

### 3.2. Moisture Content

**Figure 3:**
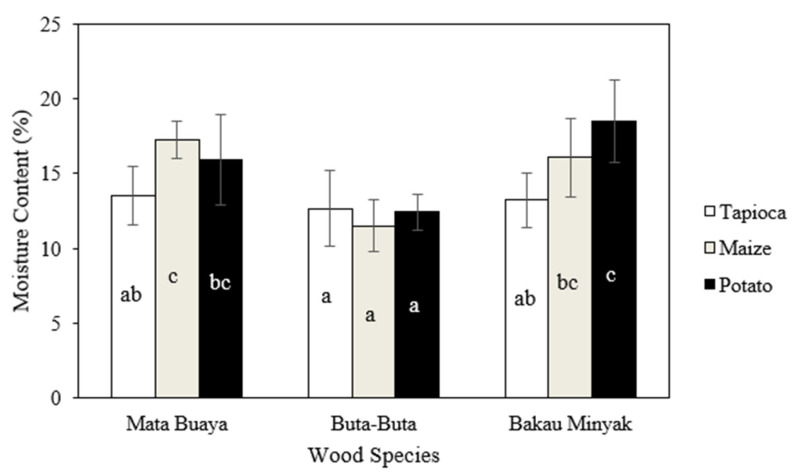
The moisture content of wood briquettes (the same letters on the bar chart are not significantly different based on the DMRT test at a 5% significance level).

The moisture content of the wood briquettes in this study ranged from 11.50−18.48%, as shown in Figure 3. Wood briquettes made of the buta-buta wood species with maize starch adhesive had the lowest moisture content, while the highest moisture content was found in wood briquettes made of bakau minyak wood species with potato starch adhesive. The moisture content of most of the wood briquettes met the ISO 17225-3:2-2020 class A2 standard and Grade A2 (KFRI), which required a maximum moisture content of 15%, except for the wood briquettes made of mata buaya and bakau minyak wood species with maize starch and potato starch adhesives. 

The analysis of variance (Table 2) revealed that the interaction between mangrove wood species and starch adhesive types, and the single factor of mangrove wood species and starch adhesive types, had a significant effect on the moisture content of the produced wood briquettes. The results of the DMRT test showed that the moisture content of briquettes made from buta-buta wood species bonded by three types of starch adhesives, as well as bakau minyak and mata buaya wood species bonded by tapioca starch adhesives, were not significantly different. However, it was significantly different from the moisture content of the wood briquettes made of bakau minyak wood species using potato starch adhesive and mata buaya wood species bonded by maize starch adhesive. 

The different types of wood raw materials in this study resulted in differences in the moisture content of the wood briquettes produced. The moisture content contained in the wood was high and varied for each different species [46]. Wood has two forms of water, namely free water and bound water. The cell cavity or lumen contains free water, which can exist as liquid or vapor. Bound water, on the other hand, is in the cell wall. The anatomical characteristics of different species of wood have different variations both macroscopically and microscopically [47]. Differences in anatomical properties affect the sizes of cavities and cell walls, thereby affecting the ability to release and absorb water molecules. The main anatomical characteristics that affect water flow during drying and the final moisture content of wood are the presence of vessels and fiber lumens, fiber walls, and pits [48]. In addition, water molecules interact closely with the chemical components of wood (cellulose, hemicellulose, and lignin) through hydrogen bonding with hydroxyl (OH) groups. The most easily accessible chemical component for hydrogen bonding with water is hemicellulose [49]. Hemicellulose is more hygroscopic than cellulose and attracts a greater number of water molecules due to its open and non-crystalline structure [50].

The use of tapioca starch adhesive in this study resulted in low moisture content of wood briquettes of the three species of mangrove wood. This is related to the differences in the basic properties of the starch adhesive used. Starch granules can absorb up to 30% of excess water by weight at room temperature. Waterschoot et al. [51] stated that the water absorption capacity per gram of starch is significantly greater for potato starch at certain temperatures than for tapioca and maize starch. Tapioca starch and maize starch have a lower water-holding capacity than potato starch. Variations in the ability to hold water can be caused by differences in the level of hydroxyl group bonding in the creation of hydrogen and covalent bonds between starch chains [52]. In addition, the particle size distribution of starch also affects water absorption. Tapioca starch had a larger particle size distribution than maize starch and potato starch (Figure 2), thus having a lower water-holding capacity. The water-holding capacity increases with decreasing starch particle size. Small starch particles have a higher solubility and increase the water absorption capacity [53,54]. These results were in accordance with the works of Handra et al. and Syarief et al. [55,56], which showed that the moisture content of briquettes bonded by sago starch adhesive, having larger particle size (57.56 µm [57]), resulted in lower moisture content. Wood briquettes with low moisture content are classified as high quality because they have higher calorific value and are easy to ignite during combustion [58].

### 3.3. Density

**Figure 4:**
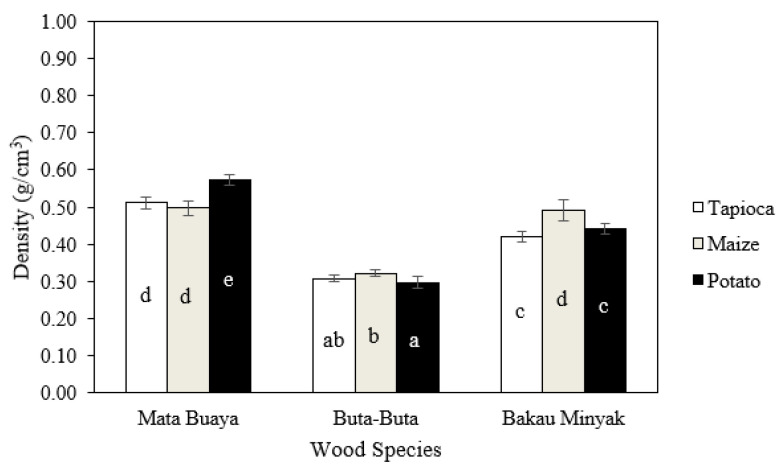
The density of wood briquettes (the same letters on the bar chart are not significantly different based on the DMRT test at a 5% significance level).

The density of the wood briquettes ranged from 0.30–0.57 g/cm^3^, as shown in Figure 4. The wood briquettes made of buta-buta with potato starch adhesive had the lowest density, while the highest density of wood briquettes was found in the wood briquettes made of mata buaya with potato starch adhesive. The density of wood briquettes in this study did not meet the ISO 17225-3:2-2020 class A2 standard or Grade A2 (KFRI), which required a minimum density of 0.9 and 1.0 g/cm^3^, respectively.

The density of the wood briquettes in this study did not meet the standards, which was caused by the hydraulic press machine used to make the briquettes having low pressure so the results were not optimal. The operating characteristics of pressure and temperature are parameters that affect the density of wood briquettes [59]. High pressure and temperature application affect the binding mechanism of wood briquettes by forming strong bonds between particles and increasing particle contact, or by reducing the distance between particles, resulting in dense and high-density wood briquettes [60]. According to Križan et al. [61], the density of wood briquettes will increase if the pressure is increased. The compaction pressure affects the formation of bonds between the pressed wood particles. The density of the resulting wood briquettes was lower than the target and standard densities due to the dimensional changes of the briquettes after compression. These changes occurred as a result of the spring-back effect. 

Dhamodaran and Afzal [62] stated that spring back was a phenomenon in which compacted powder/particle material underwent axial and radial dimensional expansion when the applied pressure was released. The residual stress appears in the form of spring back during the ejection stage of the wood briquettes from the daylight of the pressure machine. The spring back in the fibrous material after the densification process greatly increases the dimensions of the compacted biomass due to the expansion. Expansion occurred after the compressive load when pressing was released, and the wood briquettes were removed from the die, reducing their density. The condition where the elasticity of a material decreases and its plasticity increases can be caused by temperature. The application of hot temperature during wood briquette production can reduce the spring-back phenomenon but not eliminate it [63]. The low density of wood briquettes is also associated with a decrease in density due to the loss of moisture and wood particles during pressing and drying, reducing the mass per unit volume of briquettes [64]. 

The analysis of variance (Table 3) revealed that the interaction between mangrove wood species and starch adhesive types, as well as the single factor of mangrove wood species and starch adhesive types, significantly affects the density of the wood briquettes. The results of the DMRT test showed that the highest density of wood briquettes was found in mata buaya bonded by potato starch adhesive, which was significantly different from the density of all other types of wood briquettes.

Wood briquettes made of mata buaya wood species had the highest density compared to those of buta-buta and bakau minyak,. In addition, the smaller particle size distribution of mata buaya also contributes to producing wood briquettes with a higher density. Wood briquette density is related to the density of the original wood biomass used as raw material [65,66]. Križan and Mitchual et al. [67,68] stated that the density of wood briquettes made from sawdust tends to be higher when the particle sizes are smaller, as compared to larger particle sizes. In addition, using wood particles with a finer size as the raw material results in briquettes with higher density due to the larger bonding surface area. High density has a high energy content and is related to the durability of the briquettes when transported and stored. Wood briquettes with a higher density are denser, which might have an impact on their cost, handling, and transportation [69,70].

### 3.4. Compressive Strength

**Figure 5:**
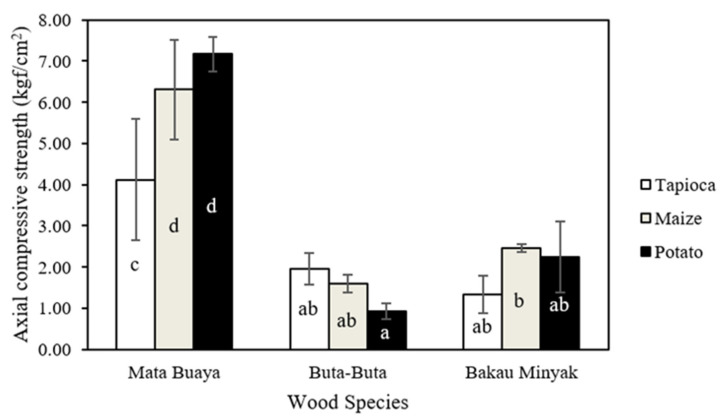
The axial compressive strength of wood briquettes (the same letters on the bar chart are not significantly different based on the DMRT test at a 5% significance level).

**Figure 6:**
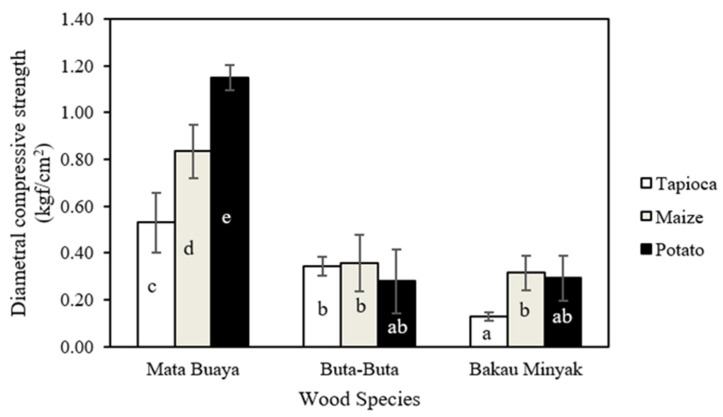
The diametral compressive strength of wood briquettes (the same letters on the bar chart are not significantly different based on the DMRT test at a 5% significance level).

The axial and diametral compressive strength values of wood briquettes are presented in Figure 5 and Figure 6. It is known that the axial compressive strength values of the wood briquettes produced in this study ranged from 0.94–7.17 kgf/cm^2^, while the value of the diametral compressive strength of wood briquettes ranged from 0.13–1.15 kgf/cm^2^. Wood briquettes made of buta-buta wood species with potato starch adhesive had the lowest axial compressive strength value, while the highest value was found in wood briquettes made of mata buaya species with potato starch adhesive. For diametral compressive strength, the lowest value was found in the wood briquettes made of bakau minyak with tapioca starch adhesive, and the highest was found in wood briquettes made of mata buaya with potato starch adhesive. In general, the axial compressive strength is higher than the diametral compressive strength [71], in line with the findings of this study. Referring to both ISO 17225-3:2-2020 class A2 standard and Grade A2 (KFRI), the compressive strength of wood briquettes (axial and diametral) is not required. 

The analysis of variance (Table 4 and Table 5) revealed that in the interaction between mangrove wood species and starch adhesive types, the single factor of mangrove wood species has a significant effect on the axial compressive strength of wood briquettes, while the single factor of starch adhesive types is vice versa. Meanwhile, the interaction between mangrove wood species and starch adhesive types, as well as the single factor of mangrove wood species and the types of starch adhesive, significantly affect the diametral compressive strength of the wood briquettes. The results of the DMRT test showed that the axial compressive strength of wood briquettes made of mata buaya wood species with potato starch adhesive was significantly different from all other types of wood briquettes, except the wood briquettes made of mata buaya bonded by maize starch adhesive. Furthermore, for the diametral compressive strength, wood briquettes made of mata buaya wood species with potato starch adhesive significantly differ from all other types of wood briquettes.

Pang et al. [72] stated that mechanical properties (compressive strength) increase with decreasing particle size. This statement is consistent with this study’s results showing that wood briquettes from the mata buaya wood species had the highest compressive strength (axial and diametral) with a smaller particle size distribution than the other types. As the particle size decreases, the compressive strength of the wood briquettes increases. Wood briquettes with a finer particle size distribution are less porous due to stronger intermolecular bonds between particles, making the particles interlock and bond together, thereby increasing the strength of the briquettes [73]. In addition, the use of starch adhesives in the production of wood briquettes for each wood species also affects its compressive strength. Bazargan et al. [71] stated that starch acts as an agent that strengthens the particles. Adding starch enhances briquettes’ strength and preserves it even after they are stored.

The wood briquettes’ density also influences the high or low compressive strength values. The wood briquettes from mata buaya wood species in this study have the highest density (Figure 4), comparable to the compressive strength values, which were also the highest compared to the wood briquettes from the buta-buta and bakau minyak wood species. This is in accordance with the statements of Jittabut and Gendek et al. [74,75], which stated that the density of briquettes correlates well with compressive strength. Briquettes with the highest density also show the highest value for compressive strength. High-quality wood briquettes have strong axial and diametral compressive strength. Both are important, as they relate to the handling and transportation of briquettes as well as their storage to prevent breakage. In this regard, packaged wood briquettes can be stacked vertically (axial) and horizontally (diametral) during transportation or storage [33].

### 3.5. Ash Content

**Figure 7:**
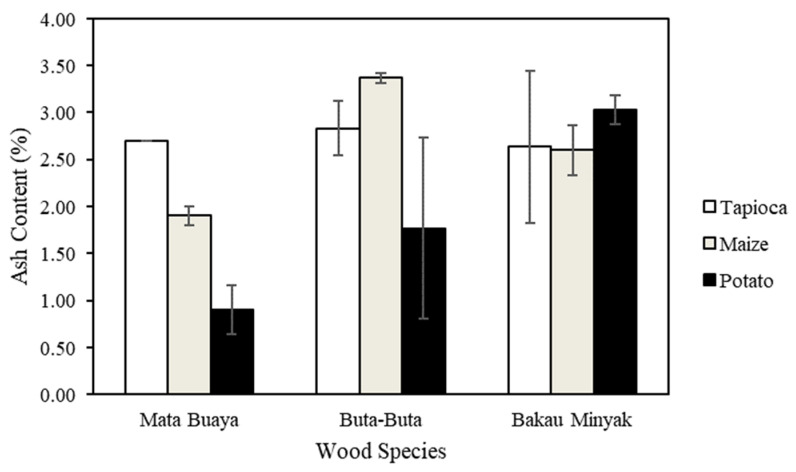
The ash content of wood briquettes.

The ash content of the wood briquettes ranged from 0.9–3.37%, as presented in Figure 7. The lowest ash content was found in wood briquettes made of mata buaya wood species bonded by potato starch adhesive, while the highest value was found in wood briquettes made of buta-buta with maize starch adhesive. The ash content of most wood briquettes met the ISO 17225-3:2-2020 class A2 standard, which required a maximum ash content of 3%, except for wood briquettes made of bakau minyak with potato starch adhesive and buta-buta with maize starch adhesive. Only wood briquettes made of mata buaya bonded by potato adhesive fulfilled the minimum criteria of ash content for Grade A2 (KFRI), which required less than 1.50%.

The ash content of wood briquettes may vary depending on the raw materials utilized [76,77]. The raw materials utilized in this study to make wood briquettes were wood branches and starch adhesives of different types. Both raw materials contribute to producing wood briquettes with varying ash content [78]. Even though the ash content of the starch was less than in the published literature (0.29% for tapioca, 0.17% for corn, and 0.31% for potato starch, respectively) [79], the ash content originating from the wood (without removal the bark) may influence the total ash content of the briquette. A previous study by Nuryawan et al. [80] showed the ash content of mangrove wood branches of the same type were relatively high, namely 2.6%, 7.3%, and 7.3%, respectively. 

High-quality wood briquettes should have low ash content. Wood briquettes with lower ash content will increase their calorific value, while higher ash content will produce dust emissions that contribute to air pollution and lower calorific value [81]. Excess ash causes problems during combustion. Ash can block the air supply, slowing down the combustion rate of wood briquettes. In other words, by making briquettes using starch adhesive, the ash content of the wood can be reduced, thereby enhancing the quality of the briquette.

### 3.6. Volatile Matter

**Figure 8:**
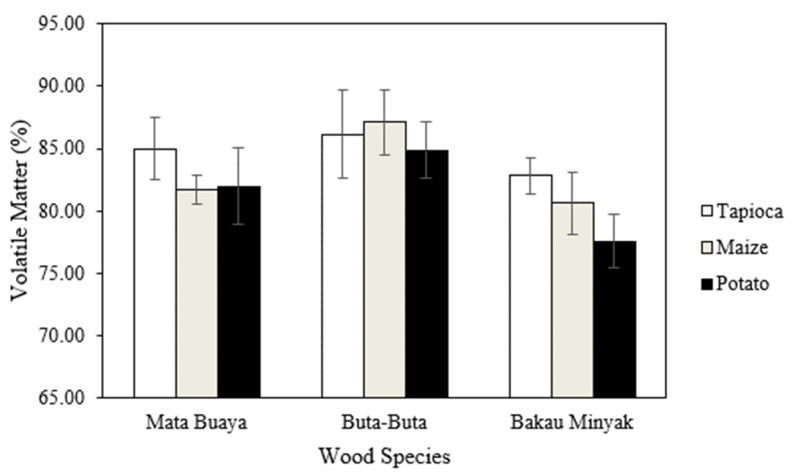
The volatile matter of wood briquettes.

The volatile matter of the wood briquettes ranged from 77.57–87.10%, as presented in Figure 8. Wood briquettes made of bakau minyak wood species bonded by potato starch adhesives had the lowest volatile matter content, while those made of buta-buta with maize starch adhesive had the highest value. Both ISO 17225-3:2-2020 class A2 standard and Grade A2 (KFRI) do not require a specific value for the volatile matter. However, the volatile matter of the wood briquettes in this study was relatively high compared to Emerhi’s findings [82], which reported the volatile matter of wood briquettes made of sawdust of three hardwood species was 72.44–77.44%. In addition, Falemara et al. [83] found the volatile matter of wood briquettes made of *Anogeissus leiocarpus* sawdust was 34.9%. 

The analysis of variance (Table 6) revealed that in the interaction between mangrove wood species and starch adhesive types there was no significant effect on the volatile matter of wood briquettes. Meanwhile, the single factor of mangrove wood species and starch adhesive type has a significant effect. DMRT test results (Table 7) show that the volatile matter of wood briquettes from the buta-buta wood species has the highest value and is significantly different from wood briquettes from the mata buaya and bakau minyak wood species. Furthermore, the use of tapioca starch adhesive in wood briquettes from the three species of mangrove wood is significantly different from the use of potato starch adhesive but not significantly different from maize starch adhesive.

The differences in the amount of volatile matter other than water contained in the raw materials for biomass and adhesives cause differences in the volatile matter in wood briquettes [84]. In almost all biomass, the volatile matter is high, generally around 70–86% of the dry biomass weight [77]. The highly volatile matter will accelerate the combustion of carbon materials and vice versa. High amounts of volatile matter in wood briquettes causes lower carbon content and more smoke generated during combustion [85]. Falemara et al. and Kpalo et al. [83,86] stated that good-quality briquettes have less than 15% volatile matter [56].

### 3.7. Fixed Carbon

**Figure 9:**
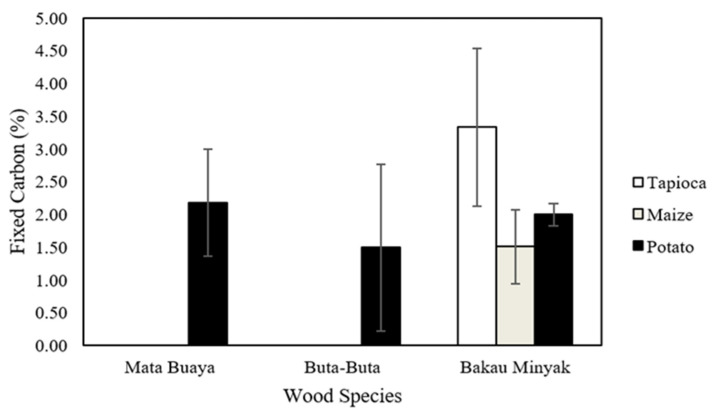
The fixed carbon of wood briquettes.

Both ISO 17225-3:2-2020 class A2 standard and Grade A2 (KFRI) do not require a fixed carbon value. Only wood briquettes bonded by potato starch adhesive and wood briquettes made of bakau minyak had these values as presented in Figure 9. Potato starch adhesive had highest solid content and bakau minyak had highest specific gravity as aforementioned in material characterization. Therefore, after heating the wood briquette samples to about 950 °C for 7 min [87], their solid residue, known as fixed carbon, were remains.

The solid residue from wood briquettes is known as fixed carbon that exists after determining volatile matter by heating a sample of wood briquettes to about 950 °C for 7 min [87]. High or low values of moisture, ash content, and volatile matter affect the value of fixed carbon [88]. It will be high if the aforesaid parameters of wood briquettes are low [89]. In this study, the volatile matter of the wood briquettes had a high value which affected the low value of the fixed carbon of the wood briquettes produced. High-quality briquettes have high fixed carbon. The higher the fixed carbon content, the higher the calorific value [88], and the better the quality of the briquettes produced. This is because a high fixed carbon content results in briquettes that produce minimal smoke during ignition and combustion.

### 3.8. Calorific Value

**Figure 10:**
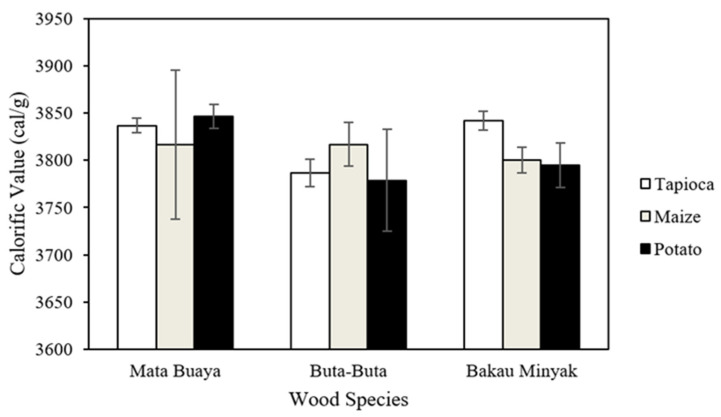
The calorific value of wood briquettes.

The calorific value of the wood briquettes ranges from 3778.78–3846.66 cal/g, as shown in Figure 10. The lowest calorific value was found in wood briquettes made of buta-buta wood species with potato starch adhesive, while the highest calorific value was found in wood briquettes made of mata buaya with potato starch adhesive. The wood briquettes’ calorific values in this study not only meet the ISO 17225-3:2-2020 class A2 standard which require a minimum calorific value of 3439 cal/g but also fulfill the Grade A2 (KFRI) criteria, which have a prerequisite calorific value of at least 3656 cal/g.

The calorific value of briquette fuel is influenced by both its moisture and ash content, which are intimately linked with the fixed carbon. High calorific briquette fuel has a low moisture and ash content [90]. In addition, density also affects the energy content of wood briquettes. High density briquettes have a high energy content [91]. Consistent with the results of this study, wood briquettes made of mata buaya wood species have the highest calorific value with lower ash content. However, there is no significant difference in calorific value among all types of wood briquettes. Based on the analysis of variance (Table 8), it was found that the interaction between mangrove wood species and starch adhesive types, as well as the single factor of mangrove wood species and starch adhesive types, had no significant effect on the calorific value of the wood briquettes. 

### 3.9. Thermogravimetric Analysis (TGA)

**Figure 11:**
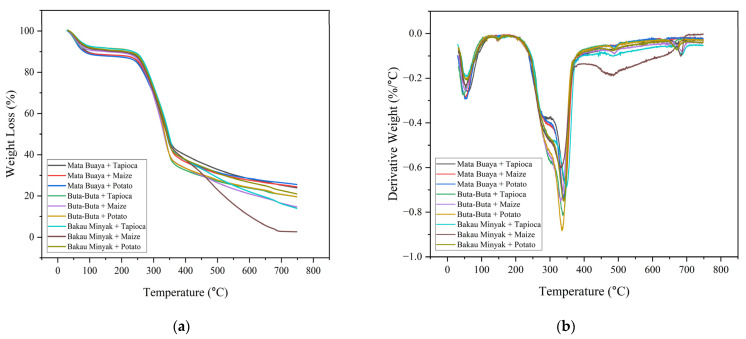
(**a**) Thermogravimetric/TG and (**b**) Derivative Thermogravimetric/DTG curves of wood briquettes.

The decrease in the weight of the wood briquettes as the temperature increased from the thermal analysis using TGA is presented in Figure 11. Based on Figure 11, the thermal degradation process of all types of wood briquettes showed a relatively similar trend that consists of three stages: the first stage occurs at the temperature range of 30–124 °C, the second stage occurs at the temperature range of 105–365 °C, and the third stage occurs at the temperature range of 362–747 °C, with different percentages of weight loss of wood briquettes as presented in Table 9. 

The weight loss of wood briquettes in the first stage ranged from 7.50–1.89%. The first stage of weight loss, which is known as the dehydration stage, is caused by the removal of moisture content. Sometimes, a small amount of volatile matter is also lost during this stage [40]. In the second stage, the weight loss of wood briquettes occurred with the highest percentage, ranging from 45.37–55.74%. The burning of volatile matter created by the degradation of hemicellulose, cellulose, and lignin occurs in the second stage. 

Maia et al. [92] stated that the highest weight loss occurs in wood briquettes at a temperature range of 200–500 °C, resulting from the decomposition of volatile materials, such as hemicellulose, cellulose, and lignin. The first component to decompose is hemicellulose, which mostly occurs at temperatures ranging from 220–315 °C. Cellulose degradation occurs at temperatures ranging from 315–400 °C. Lignin is one of the three components that are the most difficult to decompose. Lignin breakdown occurs in a wide temperature range of up to 900 °C [93]. In addition, at this stage, degradation of the starch adhesive in the wood briquettes also occurs. Gazonato et al. [94] investigated the thermal characteristics of starch and showed that weight loss related to the decomposition of amylose and amylopectin in the starch polymer occurred in the temperature range of 274–358 °C. 

Hemicellulose is thermally unstable and decomposes at lower temperatures than cellulose and lignin. This is related to the chemical structure of hemicellulose, which is amorphous and random with low strength, making it easier to decompose at lower temperatures. Meanwhile, cellulose is an extremely long unbranched polymer of glucose units with crystalline regions, making it more thermally stable, and decomposes at higher temperatures than hemicellulose [95].

The third stage is the process of decomposition of the remaining cellulose above 400 °C and the release of compounds containing oxygen and hydrogen from lignin. In this stage, the weight loss of the wood briquettes is quite high, ranging from 15.74–39.73%. According to Tsamba et al. [96], lignin breakdown starts at temperatures in the range of 160–170 °C and continues slowly up to 900 °C. Lignin contains many aromatic rings with various branches, and its chemical bond activity spans a wide range. It is highly stable and more difficult to decompose, which causes lignin decomposition to occur over a wide temperature range of up to 900 °C [97,98]. 

The variance in weight loss of each type of wood briquette in the three stages can be related to differences in the type, composition, and chemical structure of the raw materials [99], moisture content, volatile matter [100], and the differences in the strength of chemical bonds between hemicellulose, cellulose, and lignin in different biomass [101]. The chemical components of wood are related to weight loss in TGA analysis. In stage two, the weight loss is caused by the chemical decomposition of wood components (hemicellulose, cellulose, and lignin). The high weight loss in this stage is due to the decomposition of hemicellulose and cellulose in the temperature range of 220–400 °C. From the Table 9, wood briquettes made of buta-buta and bakau minyak wood species undergo higher weight loss, which is associated with their high hemicellulose and cellulose content [102].

## 4. Conclusions

The wood briquettes produced in this study mostly met the ISO 17225-3:2-2020 class A2 standard and Grade A2 (KFRI) for moisture and ash content, along with the calorific value. However, the density of wood briquettes has not met the standard. Therefore, in order to achieve the standard density, higher compaction pressure is required. Wood briquettes made of mata buaya using the three types of starch adhesives generally had better quality than all other types of wood briquettes. The interaction between mangrove wood species and the types of starch adhesive significantly affected the properties of the wood briquettes produced, except for the volatile matter and calorific value, which had no significant effect. Our findings showed that mangrove wood branches are suitable as a raw material for wood briquettes as a substitute for firewood and serve as an alternative energy source to replace fossil fuels. In general, the use of starch adhesives in the production of wood briquettes from mangrove wood branches has a similar effect, particularly with the use of maize and potato starch adhesives which have higher solid content. In the production of briquettes, the starch adhesive plays a role in binding the particles together, thereby enhancing inter-particle bonding and increasing the strength of the wood briquettes produced, both during production and storage. Wood briquettes are used for heating/cooking purposes in households and are also currently used as a fuel source in industries. Moreover, our findings can serve as a basis for further research and practical applications. For example, research on the application of various compaction pressures can be carried out to determine the optimum pressure needed to achieve better quality wood briquettes (meeting target standards).

## Figures and Tables

**Table 1:** Physical properties of mangrove wood branches.

Species	Density (g/cm^3^)	Specific Gravity
Mata buaya (*Bruguiera sexangula*)	0.90 ± 0.01	0.76 ± 0.01
Buta-buta (*Excoecaria agallocha*)	0.59 ± 0.02	0.50 ± 0.02
Bakau minyak (*Rhizophora apiculata*)	0.61 ± 0.02	0.52 ± 0.02

**Table 2:** Results of ANOVA for moisture content of wood briquettes.

	SS	df	MS	F Value	*p*-Value
Mangrove wood species (A)	123.839	2	63.420	13.220	0.000
Types of starch adhesives (B)	49.511	2	24.755	5.160	0.011
Interaction (A × B)	59.962	4	14.991	3.125	0.026

Note: SS: sum of squares; df: degrees of freedom; MS: mean square; F: F test value; *p*-value: probability value.

**Table 3:** Results of ANOVA for density of wood briquettes.

	SS	df	MS	F Value	*p*-Value
Mangrove wood species (A)	0.365	2	0.182	715.692	0.000
Types of starch adhesives (B)	0.005	2	0.003	10.044	0.000
Interaction (A × B)	0.025	4	0.006	24.323	0.000

**Table 4:** Results of ANOVA for axial compressive strength of wood briquettes.

	SS	df	MS	F Value	*p*-Value
Mangrove wood species (A)	2.182	2	1.091	8.037	0.003
Types of starch adhesives (B)	0.620	2	0.310	2.284	0.131
Interaction (A × B)	3.269	4	0.817	6.020	0.003

**Table 5:** Results of ANOVA for diametral compressive strength of wood briquettes.

	SS	df	MS	F Value	*p*-Value
Mangrove wood species (A)	1.869	2	0.935	99.738	0.000
Types of starch adhesives (B)	0.274	2	0.137	14.631	0.000
Interaction (A × B)	0.375	4	0.094	10.010	0.000

**Table 6:** Results of ANOVA for volatile matter of wood briquettes.

	SS	df	MS	F Value	*p*-Value
Mangrove wood species (A)	146.432	2	73.216	12.170	0.000
Types of starch adhesives (B)	45.353	2	22.676	3.769	0.043
Interaction (A × B)	24.035	4	6.009	0.999	0.434

**Table 7:** Results of DMRT test for single factor.

Wood Species		Types of Starch Adhesives	
Mata buaya	82.8744 b	Tapioca	86.6400 b
Buta-buta	86.0289 c	Maize	83.1322 ab
Bakau Minyak	80.3356 a	Potato	81.4667 a

The same letters on the value are not significantly different based on the DMRT test at a 5% significance level.

**Table 8:** Results of ANOVA for calorific value of wood briquettes.

	SS	df	MS	F Value	*p*-Value
Mangrove wood species (A)	4655.448	2	2327.724	1.915	0.203
Types of starch adhesives (B)	719.181	2	359.590	0.296	0.751
Interaction (A × B)	4480.629	4	1120.157	0.921	0.492

**Table 9:** The stage of thermal degradation of wood briquettes based on TGA analysis.

Types of Wood Briquettes	Stage I		Stage II		Stage III		The Residue (%)
	T (°C)	WL (%)	T (°C)	WL (%)	T (°C)	WL (%)	
Mata buaya tapioca	32–119	11.15	119–363	45.37	363–748	19.51	23.97
Mata buaya maize	30–120	11.35	120–364	48.46	364–747	15.78	24.41
Mata buaya potato	30–124	11.89	124–362	46.83	362–745	15.74	25.53
Buta-buta tapioca	32–106	9.58	106–363	54.89	363–747	16.23	19.30
Buta-buta maize	33–106	9.48	106–363	53.77	363–747	22.14	14.61
Buta-buta potato	32–108	7.88	108–364	55.75	364–747	16.98	19.39
Bakau minyak tapioca	30–105	7.50	105–365	51.05	365–746	27.41	14.04
Bakau minyak maize	33–108	8.71	108–363	48.94	363–745	39.73	2.62
Bakau minyak potato	32–110	8.33	110–362	50.45	362–747	20.21	21.02

Note: T: temperature; WL: weight loss.

## Data Availability

The data presented in this study are available on request from the corresponding author.
